# Correction: Weng et al. New International Association for the Study of Lung Cancer (IASLC) Pathology Committee Grading System for the Prognostic Outcome of Advanced Lung Adenocarcinoma. *Cancers* 2020, *12*, 3426

**DOI:** 10.3390/cancers13164024

**Published:** 2021-08-10

**Authors:** Ching-Fu Weng, Chi-Jung Huang, Shih-Hung Huang, Mei-Hsuan Wu, Ailun Heather Tseng, Yung-Chuan Sung, Henry Hsin-Chung Lee, Thai-Yen Ling

**Affiliations:** 1 Division of Pulmonary Medicine, Department of Internal Medicine, Hsinchu Cathay General Hospital, Hsinchu 300, Taiwan; cgh18497@cgh.org.tw; 2 Department and Graduate Institute of Pharmacology, National Taiwan University, Taipei 100, Taiwan; 3 Medical Research Center, Cathay General Hospital, Taipei 106, Taiwan; aaronhuang@cgh.org.tw; 4 Department of Biochemistry, National Defense Medical Center, Taipei 114, Taiwan; 5 School of Medicine, Fu Jen Catholic University, New Taipei 242, Taiwan; 6 Division of Pathology, Cathay General Hospital, Taipei 106, Taiwan; ja68@cgh.org.tw; 7 Teaching and Research Center, Hsinchu Cathay General Hospital, Hsinchu 300, Taiwan; markicoo@cgh.org.tw; 8 Department of Biomedical Sciences and Engineering, National Central University, Taoyuan 320, Taiwan; c8500@cgh.org.tw; 9 Division of Hematology/Oncology, Department of Internal Medicine, Cathay General Hospital, Taipei 106, Taiwan; cgh06496@cgh.org.tw; 10 Department of Surgery, Hsinchu Cathay General Hospital, Hsinchu 300, Taiwan; 11 Graduate Institute of Translational and Interdisciplinary Medicine, College of Health Sciences and Technology, National Central University, Taoyuan 320, Taiwan

The authors would like to make a correction to their published paper [1].

There was a mistake in the original version of the article in Figures 1 and 2. We found that Figures 1 and 2 were misplaced in the article.

They should be replaced with the following Figure 1 and Figure 2:

**Figure 1:**
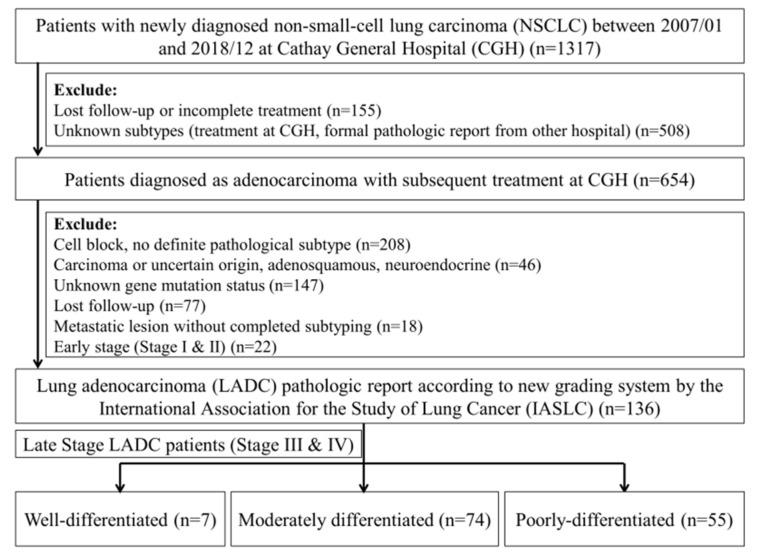
Selection criteria for the subjects.

**Figure 2:**
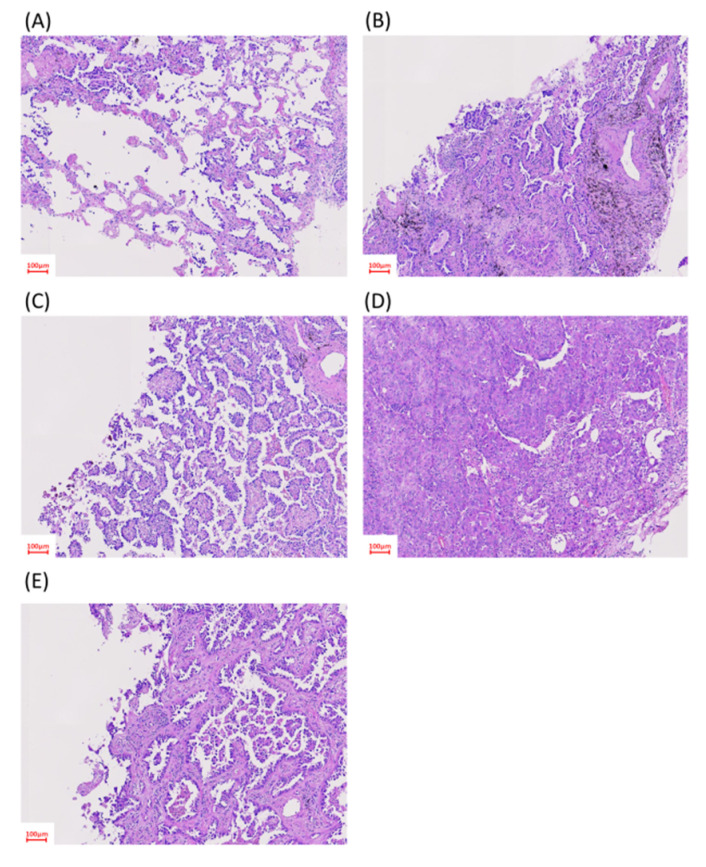
Histological patterns of lung adenocarcinoma. Representative hematoxylin and eosin-stained biopsy specimens: (**A**) lepidic-predominant pattern; (**B**) acinar-predominant pattern; (**C**) papillary-predominant pattern; (**D**) solid-predominant pattern; and (**E**) micropapillary-predominant pattern.

The authors apologize for any inconvenience caused and state that the scientific conclusions are unaffected. The original article has been updated.
