# Supplementary figures and images for: New International Association for the Study of Lung Cancer (IASLC) Pathology Committee Grading System for the Prognostic Outcome of Advanced Lung Adenocarcinoma
Source: Cancers (Basel). 2020 Nov 18;12(11):3426. doi: 10.3390/cancers12113426 (PMC7698816; doi:10.3390/cancers12113426)

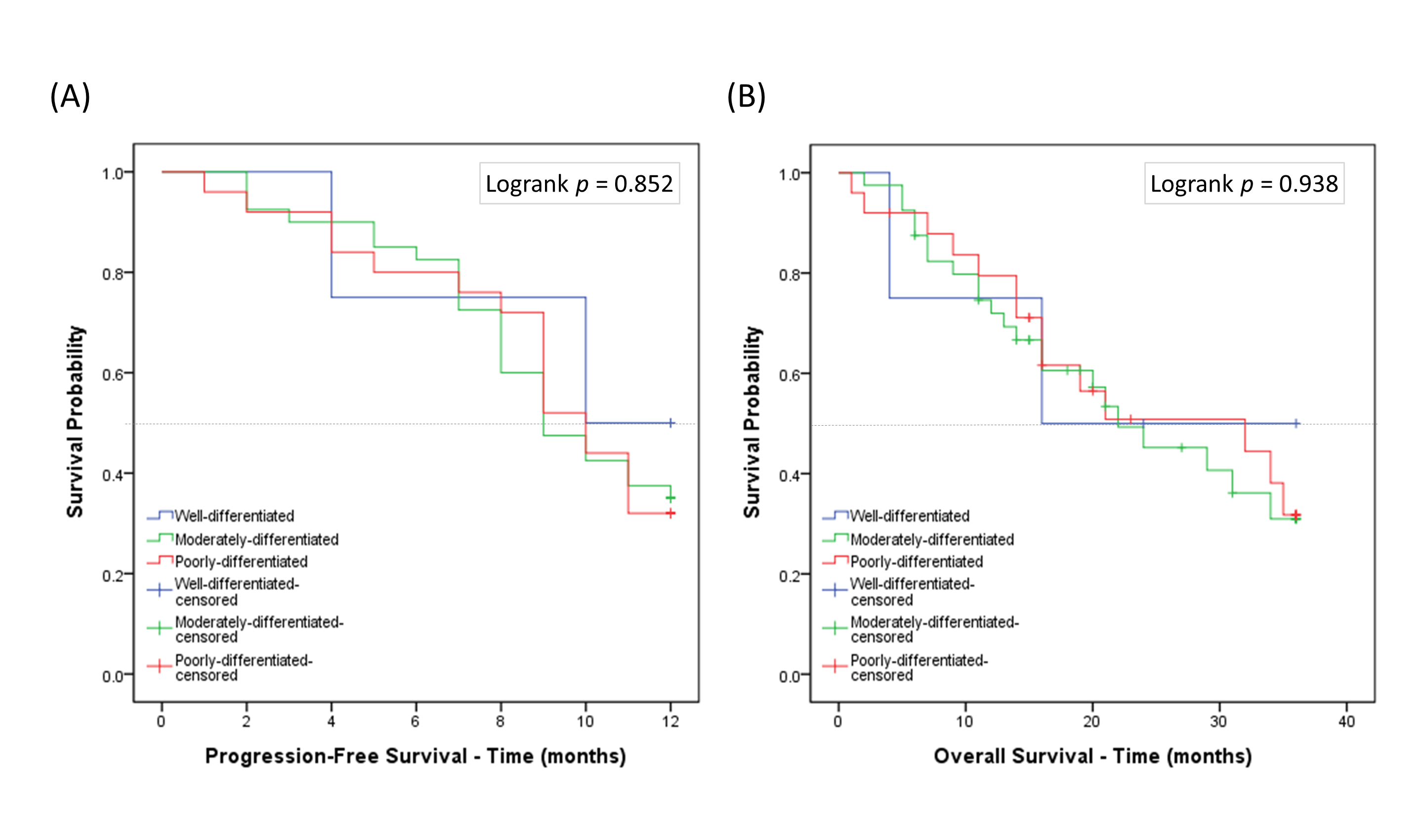

Supplement: Supplementary file 1 [file cancers-12-03426-s001.zip › FigureS1.TIF]

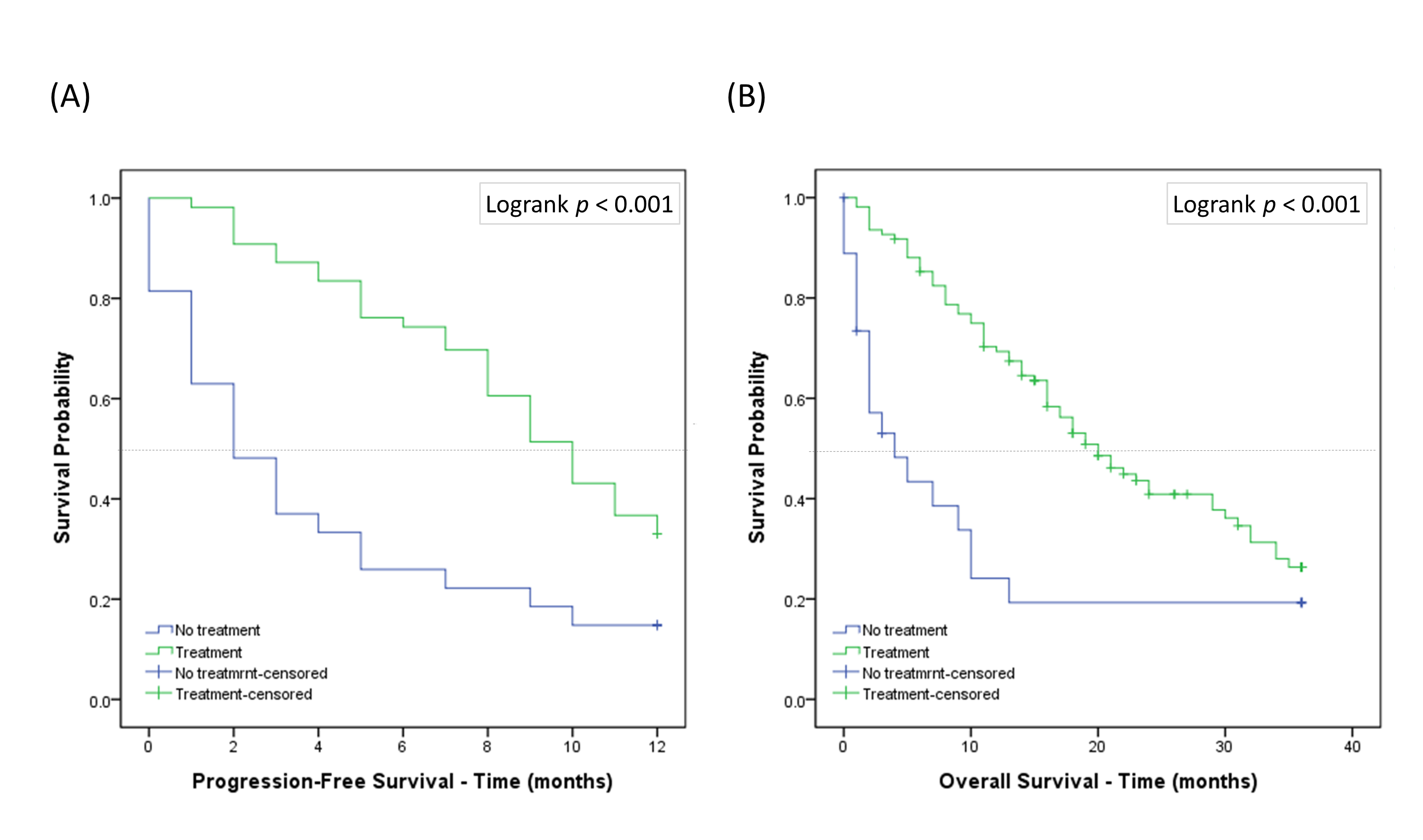

Supplement: Supplementary file 1 [file cancers-12-03426-s001.zip › FigureS2.tif]

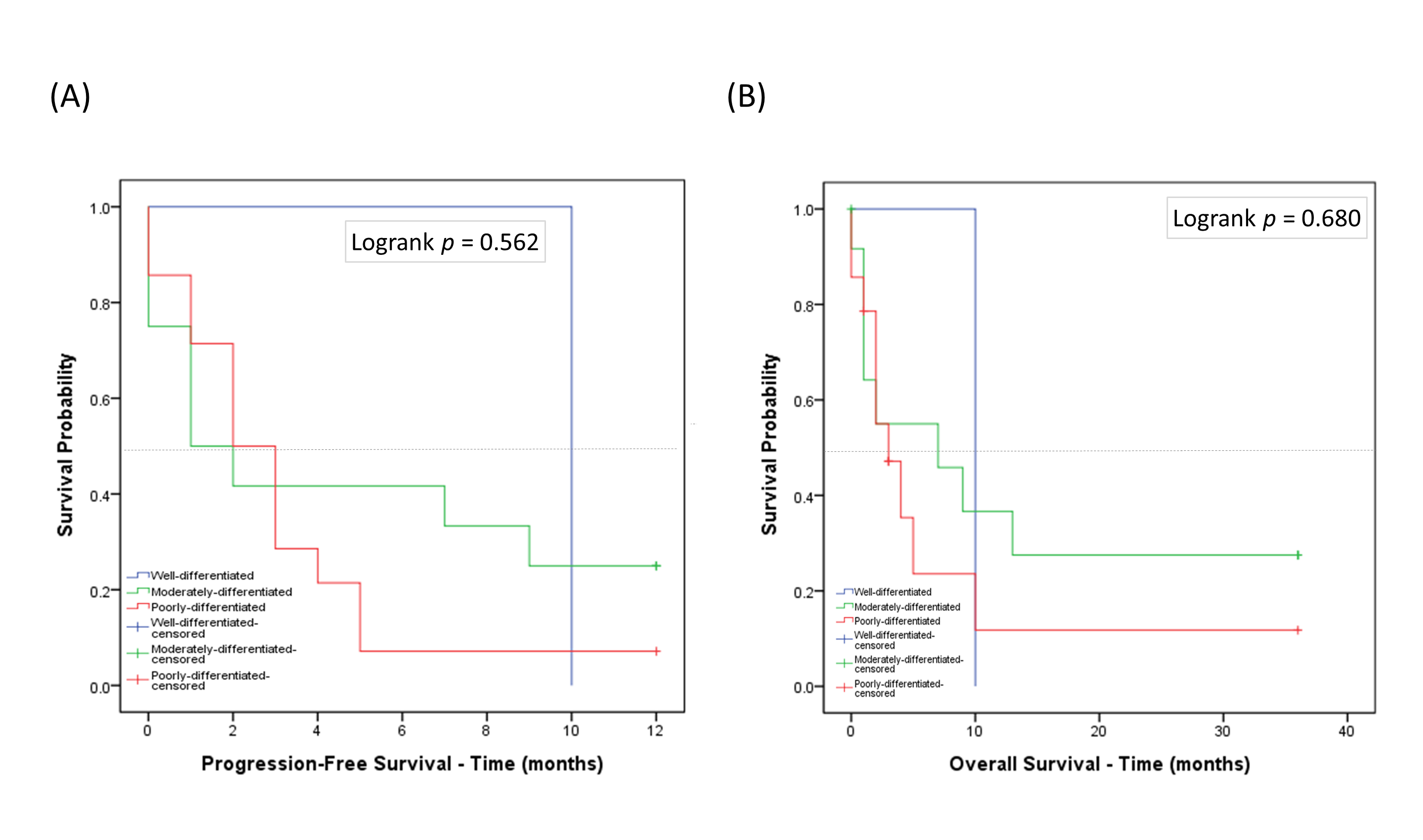

Supplement: Supplementary file 1 [file cancers-12-03426-s001.zip › FigureS3.tif]

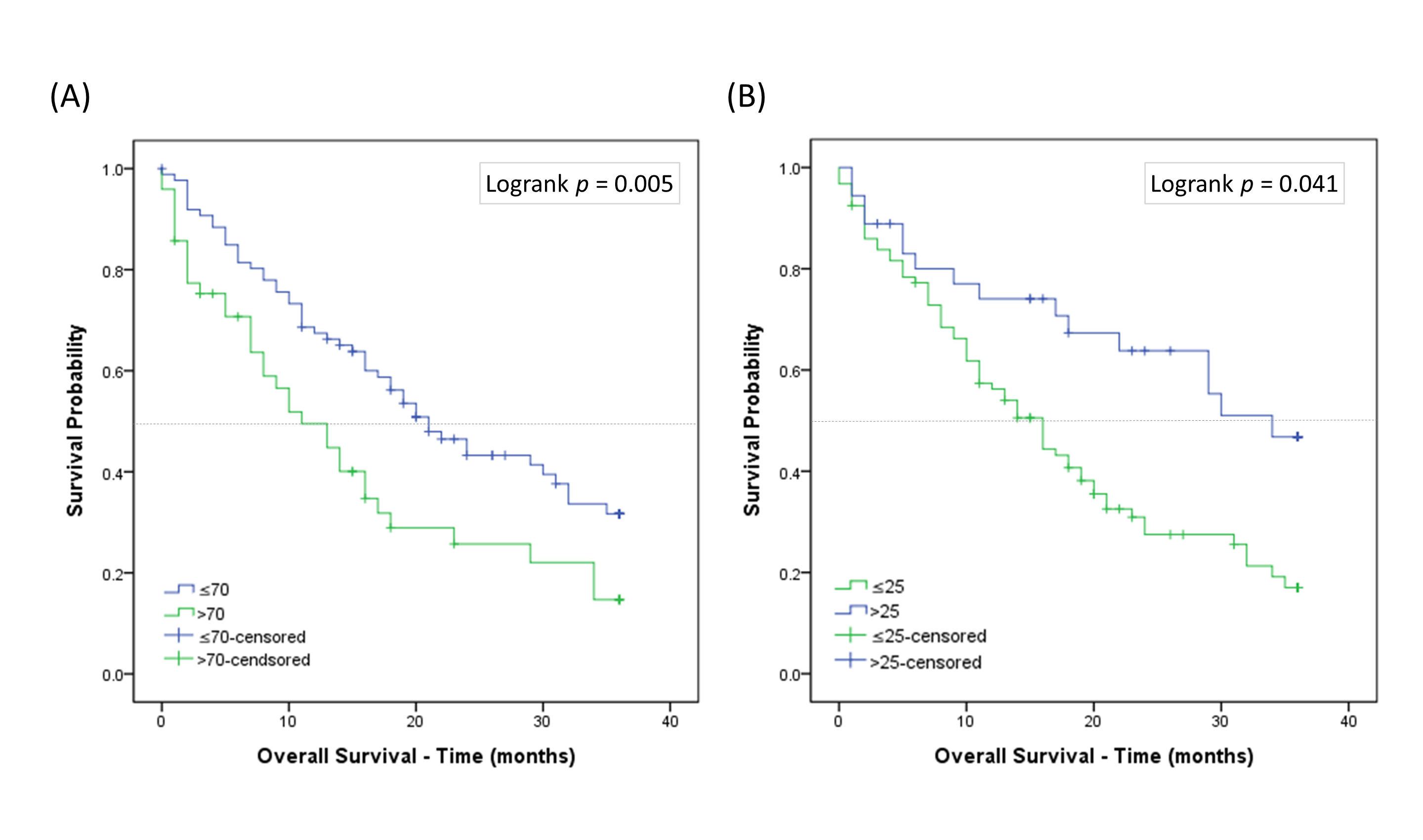

Supplement: Supplementary file 1 [file cancers-12-03426-s001.zip › FigureS4.TIF]
